
JOURNAL INFORMATION
==============================
NLM Title Abbreviation: Behav Neurol
Journal ID: Behav Neurol
NLM Journal ID: 8914585
Journal ID: BN

Behavioural Neurology

ISSN: 0953-4180
EISSN: 1875-8584
Publisher: Wiley

ARTICLE INFORMATION
==============================
PMCID: PMC5215756
PMID: 23242353
DOI: 10.3233/BEN-120301
Article ID: 835253
Article version: 1
Subjects: Other

Thalamic Deep Brain Stimulation for Tourette Syndrome

Ackermans L. 1
Neuner I. 2
Temel Y. 1 3
Duits A. 4 5
Kuhn J. 6
Visser-Vandewalle V. 7 *
1 Department of Neurosurgery Maastricht University Medical Centre Maastricht The Netherlands
2 Department of Psychiatry and Psychotherapy RWTH Aachen University Aachen Germany
3 European Graduate School of Neuroscience (EURON) Faculty of Health, Medicine and Life Science Maastricht The Netherlands
4 Department of Psychiatry and Psychology Maastricht University Medical Centre Maastricht The Netherlands
5 School of Mental Health and Neuroscience (MHENS) Mondriaan Zorggroep Maastricht The Netherlands
6 Department of Psychiatry and Psychotherapy University of Cologne Cologne Germany
7 Department of Stereotactic and Functional Neurosurgery University of Cologne Cologne Germany
*V. Visser-Vandewalle: veerle.visser-vandewalle@uk-koeln.de
Print publication date: 2013
Volume: 27
Issue: 1
First page: 133
Last page: 138
Received 2012 Dec 10; Accepted 2012 Dec 10
Copyright: Copyright © 2013 Hindawi Publishing Corporation and the authors.
Copyright year: 2013
License: This is an open access article distributed under the Creative Commons Attribution License, which permits unrestricted use, distribution, and reproduction in any medium, provided the original work is properly cited.
License URL: https://creativecommons.org/licenses/by/3.0/

Keywords: Tourette syndrome, thalamus, deep brain stimulation

==============================
Behavioural Neurology 27 (2013) 133–138
DOI 10.3233/BEN-120301
IOS Press

### Thalamic deep brain stimulation for Tourette syndrome

L. Ackermans[a], I. Neuner[b], Y. Temel[a,c], A. Duits[d,e], J. Kuhn[f] and V. Visser-Vandewalle[g,*]
[a]*Department of Neurosurgery, Maastricht University Medical Centre, Maastricht, The Netherlands*
[b]*Department of Psychiatry and Psychotherapy, RWTH Aachen University, Aachen, Germany*
[c]*European Graduate School of Neuroscience (EURON), Faculty of Health, Medicine and Life Science, Maastricht, The Netherlands*
[d]*Department of Psychiatry and Psychology, Maastricht University Medical Centre, Maastricht, The Netherlands*
[e]*School of Mental Health and Neuroscience (MHENS), Mondriaan Zorggroep, Maastricht, The Netherlands*
[f]*Department of Psychiatry and Psychotherapy, University of Cologne, Cologne, Germany*
[g]*Department of Stereotactic and Functional Neurosurgery, University of Cologne, Cologne, Germany*

Keywords: Tourette syndrome, thalamus, deep brain stimulation

### 1. Introduction

Tourette syndrome (TS) is a complex chronic neuropsychiatric disorder characterized by motor and vocal tics. Motor tics are sudden, repetitive, stereotyped movements such as eye blinking, facial twitching, and head or shoulder movements, whereas vocal or phonic tics are sounds produced by moving air through the nose, mouth, or throat (e.g. coughing and throat clearing) as well as repeating syllables, words, or phrases [1]. TS typically has an onset in early childhood, and boys are more commonly affected than girls. Symptoms usually start with transient bouts of simple motor tics. Tics can become more "complex" in nature and appear to be purposeful. A fleeting feeling of relief often follows performance of a tic or a series of tics [2, 3]. Tics typically follow a waxing and waning pattern of severity, intensity, and frequency [4]. Tic severity usually peaks between 8 and 12 years of age, with many patients showing a marked reduction in severity by the end of adolescence [5–7]. Approximately 20% of children with TS continue to experience a moderate level of impairment of global functioning by the age of 20 years [7]. TS alone is the exception rather than the rule. Attention deficit/hyperactivity disorder (ADHD) and obsessive-compulsive behaviour (OCB) are the most common co-morbidities. The presence of these co-morbidities can add another layer of complexity, which may make it more difficult to develop a treatment plan that not only addresses the tics, but also the co-occurring disorders.

TS might be interpreted as an overactive abnormal neural activity of both the sensimotor and the limbic circuits, involving multiple outputs, of the basal ganglia [8].

### 2. Treatment of TS

Frequently, TS is found to be a self-limiting disorder, whereas in a small proportion of patients the tics continue into adult life and require long-term behavioural or drug treatment. Behavioural and drug treatment may provide temporary control of symptoms but certain patients prove to be medically untreatable or experience unbearable side effects from the medication. For these patients, surgery may be an option.

In the past, various attempts have been made to treat patients suffering from TS through neurosurgical abla-

*Corresponding author: Veerle Visser-Vandewalle, Department of Stereotactic and Functional Neurosurgery, University of Cologne, Cologne, Germany. E-mail: veerle.visser-vandewalle@uk-koeln.de.

ISSN 0953-4180/13/$27.50 © 2013 – IOS Press and the authors. All rights reserved tive procedures [9]. The target sites have been diverse including the frontal lobe (prefrontal lobotomy and bimedial frontal leucotomy), the limbic system (limbic leucotomy and anterior cingulotomy), the thalamus and the cerebellum. The results have often been unsatisfactory or major side effects have occurred such as hemiplegia or dystonia. Hassler and Dieckmann reported on the beneficial effects of lesioning the intralaminar and midline thalamic nuclei in patients suffering from TS, and, in patients with facial tics, also the nucleus ventro-oralis internus (Voi) [10].

Deep brain stimulation (DBS) has been introduced in the field of neuropsychiatry to modulate neuronal activity in the same areas as those targeted for lesioning in the past, but in a reversible way.

The difficulty was that Hassler made up to ten coagulations in the intralaminar and medial thalamic nuclei, so a strategic point had to be found so that as many of the nuclei targeted by Hassler could be stimulated by one electrode for each hemisphere. This strategic point was found, studying the Schaltenbrand-Wahren atlas [11], on a coronary slice at 4 mm posterior to the midpoint of the line connecting the anterior commissure (AC) with the posterior commissure (PC) and 5 mm lateral to the AC-PC line, and the dorsolateral plane, at the level of AC-PC. The first target for DBS, the medial part of the thalamus at the crosspoint of the centromedian nucleus (Cm), the substantia periventricularis (SPv) and the nucleus ventro-oralis internus (Voi), has been described by Vandewalle in 1999 [12]. The results of thalamic DBS in the first 3 Tourette patients have been described in 2003 [13].

### 3. Targets

In the last ten years, more than 90 patients with TS have been reported to be treated with DBS in the literature, with ten different brain targets, including the Cm-Spv-Voi cross point of the thalamus [12–19]. The group of Servello and Porta [20–22] targeted the same area but at a point 2 mm more anteriorly. And one case is described with DBS of the dorsomedial thalamus [23,24]. Besides the thalamus, the globus pallidus externus (GPe) [25] and both the ventroposterolateral motor, as well as the anteromedial limbic part of the globus pallidus internus (GPi) have been targeted for DBS in refractory TS [26–33]. Also the nucleus accumbens [34–38], and internal capsule [16,37,38], have been described, mostly in TS patients also suffering from OCs. Finally, in a patient suffering from both Parkinson's disease and tics, there was an improvement of tics after DBS of the subthalamic nucleus [39].

More detailed information about the targets and results are described in an excellent review by Piedad et al. [40].

The general rationale for modulating these areas is based on the assumption that tics and associated behavioural disorders are related to a dysfunction of the cortico-basal ganglia-thalamocortical circuitry. Focal disruption of different functional striatal territories results in abnormal activation of neocortical motor and non-motor areas, producing repeated stereotyped movements and abnormal behaviour [41].

### *3.1. Thalamic DBS*

After the promising results of DBS in the first TS patient described by Vandewalle et al. [12], the same group reported on the beneficial effects of DBS of the same target in three patients in [13]. The authors stated that stimulation of the nucleus ventro-oralis internus would lead to diminished motor and vocal tics through inhibiting projections to the facial parts of the premotor (and motor) cortex. Stimulation of the intralaminar nuclei would reduce the activity of the dorsal, sensorimotor parts of the striatum, while stimulation of the midline thalamic nuclei would reduce activity in the ventral, limbic striatum. Almost 80 patients received thalamic DBS for intractable TS, although within the thalamic target there has been some variety. However, sample sizes are small, with the majority of studies being single case reports, there have only been a limited number of studies of thalamic DBS in TS with an accurate methodological design [15,19].

Visser-Vandewalle reported in [12,13] the results of the first three Tourette patients. There was not only a good effect on tics but also on associated behavioural disorders, like obsessive-compulsive behaviour (OCB) and self-injurious behaviour (SIB). With a follow-up period of respectively 5 years, 1 year and 8 months, there was not only a good effect on tics (with a tic reduction of resp. 90%, 72% and 83% with stimulation on compared with the stimulation off condition), but also on associated behavioural disorders such as obsessive-compulsive disorder (OCD) or self-injurious behavior (SIB). Stimulation induced side effects consisted of drowsiness, and changes in sexual functioning. The long-term outcomes of the first and second patient were described in a recent report by the same authors [42]. Tic improvement observed at 5 years in patient 1 (90.1%) was maintained at 10 years (92.6%).

In patient 2, the tic improvement at 8 months (82%) was slightly decreased at 6 years (78%).

Maciunas et al. described in [15] the effects of DBS in five patients with intractable TS, with a follow-up of three months. They used the same target as described by Vandewalle et al. in 1999. After the first four weeks postoperatively, randomized double-blinded assessments showed a statistically significant reduction in motor and vocal tics. At three months, open label assessments showed in three out of five patients an average tic reduction of 50%. The secondary outcome measures anxiety, depression and OCD showed a trend towards improvement. In one patient, a psychotic event was described as an unwanted stimulation-related effect. Bajwa et al. [16] found a good effect of DBS of this thalamic target in a 50 years old patient suffering from TS with OCD and SIB. After 24 months, stimulation resulted in a 66% improvement on the Yale Global Tic Severity Scale (YGTSS), and a 76% reduction of the YBOCS. Idris presented one case with bilateral cortical hematomas after thalamic DBS, with a short note that complex motor and vocal tics improved [17]. In one patient only mild improvement was seen after DBS of the CM/SPv/VOi crosspoint [18]. Most recently, Ackermans et al. presented a double-blind randomized clinical trial of six TS patients with 49% improvement on tics and no significant difference in associated behavior [19].

In 2008, Servello et al. [20] reported on the beneficial effects of DBS in 18 patients with TS, with the target being located 2 mm more anteriorly than the one from Vandewalle et al. After an unblinded follow op from 3–17 months, there was a tic improvement varying between 24 and 79% in 15 out of 18 patients. The authors also described a good effect on behavioural disorders. As a stimulation-induced negative side effect, temporary disturbances of oculomotion were mentioned. Fifteen of these 18 patients were followed for 2 years and showed 52% tic improvement at this long-term follow-up [21].

Additionally, this same group of Servello et al. reported on 36 Tourette patients with different brain targets. In total 19 patients were followed for two years after thalamic stimulation of the CM-Pf/Vo with a 54.2% tic reduction [22].

Finally, the beneficial effect of DBS of the dorsomedial nucleus of the thalamus has been described by Vernaleken et al. [24,25] in one patient. After unsuccessful DBS of the GPi, the electrodes were removed and two electrodes were implanted in the Cm-Pf complex of the thalamus. The patient showed the highest benefit with stimulation of the most dorsal contacts, which were located in the dorsomedial nucleus, with a 36% improvement of tics.

### 4. Clinical and surgical evaluation

### *4.1. Patient selection and surgical procedure*

Careful patient selection is absolutely mandatory for DBS in TS [43]. The TS patients considered for DBS should comprise only very severe cases in whom tics are life threatening, result in physical disability or lead to functional impairment and cause significant impairment in quality of life. And in who have already fruitlessly received standard therapies. Several papers providing guidelines for DBS in TS [43–45] have addressed this.

Surgeons should have substantial experience in DBS treatment of movement disorders to enhance efficacy and minimize complications. The technique of DBS applied to TS is in broad lines similar to the one used for more classic indications. The target for TS, such as the nuclei of the medial part of the thalamus, is mostly invisible with current imaging techniques. Moreover, TS patients might pull themselves out of the stereotactic frame because of the high ratio of motor tics occurring in the head region. One solution would be to operate with the patient being under general anaesthesia. Because of the uncertainty of the ideal target and the importance of intra-operative findings, the patient should be cooperative during surgery. Sedating the patient to obtain tic suppression with maintenance of the possibility to communicate with the patients is preferable. The patients can be sedated with a combination of lormetazepam and clonidine [12], or with a Propofol Target Controlled Infusion [42], sufficiently reducing the tics and their implications for the stereotactic procedure. At the same time the patient can be interrogated so that acute negative stimulation-induced side effects can be detected and the position of the electrode adapted.

### *4.2. Peri- and post-operative evaluation*

It is of paramount importance that for all TS patients treated with DBS, the exact location of the electrode is precisely determined and all effects are meticulously described. A more comprehensive survey of guidelines for the peri-operative assessment of the effects of DBS in TS can be found elsewhere [43].

First, the execution of DBS should be restricted to neurosurgical units experienced in the DBS treatment of movement disorders with established collaborations with neurological and psychiatric departments specialized in the diagnosis and treatment of TS. For the assessment of clinical effects, a description of the effect on tics, on associated behavioural disorders, the stimulation-induced side effects, and complications, are mandatory. The most commonly used scale for tic rating is the Yale Global Tic Severity Scale (YGTSS) [46]. For a more objective evaluation, the patient should also be recorded on videotape with and without stimulation. The tics should be rated on these tapes by two independent investigators. Ideally the patient and investigator are blinded to the status of the stimulation. A careful psychiatric and neuropsychological evaluation should be performed at regular intervals. The clinical effects should be correlated to the exact position of the electrode. The most prudent approach is to perform a CT-scan postoperatively, and fuse these images with preoperative MR-images. Only if these prerequisites are fulfilled and a maximum of data is exchanged between centres, the optimal target can be established.

### 5. Complications and side effects after thalamic DBS

Two major complications have been described, consisting of a small intracranial haemorrhage in one patient resulting in vertical gaze palsy during six months and subjective changes in the velocity of upward gaze afterwards [47]. One patient had intracerebral hematomas located around both electrodes [17].

The group of Servello [22] reported on the need for repositioning of the two leads in one patient and removal of the DBS implant due to an infection along the extension cables and pulsgenerator in another patient. Two other patients required revision of surgical wounds along the extension cables because of diastasis, and two patients required surgical revision of the pulsgenerator because of infection. Finally hardware failure with monolateral extension cable rupture was documented in one patient.

Six Tourette patients have been extensively followed and described by Ackermans et al. [19]. Some side effects were noticed, especially lack of energy resulting in restriction in their daily activities and visual changes. One patient of the randomized controlled trial reported vertical gaze difficulty after thalamic DBS due to a haemorrhage at the end of the left electrode [47]. Because the thalamic target for DBS is located near the nuclei responsible for vertical gaze, trajectory planning and electrode positioning should be performed with a special focus on vertical eye movements and with extreme accuracy and caution. Aside from the proven vertical gaze disturbances, all the other included patients reported visual problems. This may not be due to ocular abnormalities but to visual processing problems. Visual processing difficulties are frequently present in TS and are thought to be most reflective of the underlying frontostriatal dysfunction [48]. Previous studies examining saccades in TS have reported however conflicting results [48]. Servello et al. [20] also reported on stimulation induced subjective vertigo, blurring of vision and upward ocular deviation after thalamic DBS. In this study however these symptoms appeared to be transient.

### 6. Conclusion

Given the many different targets used for DBS in TS, and the small number of patients suffering form the intractable syndrome, continuous exchange of clinical experience, and an on-going evaluation is important. A uniform approach with standard inclusion criteria and outcome measures is warranted to find out which is the most optimal target, or whether "tailored" targeting is needed, with a specific target for a specific subtype of patients, as also suggested by Porta et al. [49].

Given the consequences of TS for social, familial and professional life, patients have to deal with many challenges after surgery. Anticipating on these postoperative changes prior to surgery will be helpful to assist patients and their families in benefiting from tic reduction and maximizing the overall outcome and success of surgery.

The results of thalamic DBS in TS prove that the technique is effective in the treatment of carefully selected patients. Because of the occurrence of stimulation related side effects the search for the most optimal target is still going on. Patients treated with DBS will need long-term follow-up in order to answer questions regarding long-term efficacy, and outcome. In addition, the phenotypic variability of TS patients and various co-morbidities make it even more difficult to evaluate exactly what circuits should be modulated depending on the predominant clinical features. Given the complexity of TS and difficulties adhering to crossover protocols, future studies should consider delayed therapy or randomization to ‘off’ or ‘on’ groups rather than a crossover design.

Determination of the optimal surgical target and stimulation parameters will require close multicentre collaboration and standardized methods for postoperative evaluations. Other questions still to be addressed are whether tolerance or hardware failure would play a role. Therefore a prospective, multicenter double-blinded study to evaluate the effects of DBS in selected Tourette patients would be the most ideal approach.

### References

[1] Mink JW. Basal ganglia dysfunction in Tourette’s syndrome: a new hypothesis. Pediatric Neurology 2001; 25: 190-98.

[2] Leckman JF, Walker DE, Cohen DJ. Premonitory urges in Tourette’s syndrome. Am J Psychiatry 1993; 150: 98-102.

[3] Woods DW, Piacentini J, Himle MB, Chang S. Premonitory Urge for Tics Scale (PUTS): Initial psychometric results and examination of the premonitory urge phenomenon in youths with Tic disorders. J Devel Behav Pediatr 2005; 26: 397-403.

[4] Leckman JF. Tourette’s syndrome. Lancet 2002; 360: 1577-86.

[5] Leckman JF, Zhang H, Vitale A, Lahnin F, Lynch K, Bondi C, Kim Y-S, Peterson BS. Course of tic severity in Tourette syndrome: The first two decades. Pediatrics 1998; 102: 14-9.

[6] Coffey BJ, Biederman J, Geller D, Frazier J, Spencer T, Doyle R, Gianini L, Small A, Frisone DF, Magovcevic M, Stein N, Faraone SV. Reexamining tic persistence and tic-associated impairment in Tourette’s disorder: Findings from a naturalistic follow-up study. J Nervous Mental Dis 2004; 192: 776-80.

[7] Bloch MH, Peterson BS, Scahill L, Otka J, Katsovich L, Zhang H, Leckman JF. Adulthood outcome of tic and obsessive-compulsive symptom severity in children with Tourette syndrome. Arch Pediatr Adolesc Med 2006; 160: 65-9.

[8] Babel TB, Warnke PC, Ostertag CB. Immediate and long term outcome after infrathalamic and thalamic lesioning for intractable Tourette’s syndrome. J Neurol Neurosurg Psychiatry 2001; 70: 666-71.

[9] Temel Y and Visser-Vandewalle V. Surgery in Tourette syndrome. Mov Disord 2004; 19: 3-14.

[10] Hassler R, Dieckmann G. Traitement stéréotaxique des tics et cris inarticulés ou coprolaliques considérés comme phénomène d’obsession motrice au cours de la maladie de Gilles de la Tourette. Rev Neurol Paris 1970; 123: 89-100.

[11] Schaltenbrand G, Wahren W (1977). Atlas for stereotaxy of the human brain. $2^{nd}$ ed Stuttgart, Thieme.

[12] Vandewalle V, van der Linden C, Groenewegen HJ, Caemaert J. Stereotactic treatment of Gilles de la Tourette syndrome by high frequency stimulation of the thalamus: case report. Lancet 1999; 353: 724.

[13] Visser-Vandewalle V, Temel Y, Boon P, Vreeling F, Colle H, Hoogland G, Groenewegen H, van der Linden Ch. Chronic bilateral thalamic stimulation: A new therapeutic approach in intractable Tourette syndrome. J Neurosurg 2003; 99: 1094-1100.

[14] Bawja RJ, de Lotbiniere AJ, King RA, Jabbari B, Quatrano S, Kunze K, Scahill L, Leckman JF. Deep brain stimulation in Tourette’s syndrome. Mov Disord 2007; 22: 1346-50.

[15] Maciunas RJ, Maddux BN, Riley DE, Whitney CM, Schoenberg MR, Ogrocki PJ, Albert JM, Gould DJ. Prospective randomised double-blind trial of bilateral thalamic deep brain stimulation in adults with Tourette syndrome. J Neurosurg 2007; 107: 1004-14.

[16] Shields DC, Cheng ML, Flaherty AW, Gale JT, Eskandar EN. Microelectrode-guided deep brain stimulation for Tourette syndrome: Within-subject comparison of different stimulation sites. Stereotact Funct Neurosurg. 2008; 86: 87-91.

[17] Idris Z, Ghani AR, Mar W, Bhaskar S, Wan Hassan WN, Tharakan J, Abdullah JM, Omar J, Abass S, Hussin S, Abdullah WZ. Intracerebral haematomas after deep brain stimulation surgery in a patient with Tourette syndrome and low factor XIIIA activity. J Clin Neurosci 2010: 17; 1343-4.

[18] Janik P, Hozaria H, Mandat T. Deep brain stimulation of CM-Spv-Voi complex for alleviating symptoms of Tourette syndrome. Parkinsonism Relat Disord 2009: 15(S2); S30.

[19] Ackermans L, Duits A, van der Linden C, Tijssen M, Schruers K, Temel Y, Kleijer M, Nederveen P, Bruggeman R, Tromp S, van Kranen-Mastenbroek V, Kingma H, Cath D, Visser-Vandewalle V. Double-blind clinical trial of thalamic stimulation in patients with Tourette syndrome. Brain 2011; 134: 832-44.

[20] Servello D, Porta M, Sassi M, Brambilla A, Robertson MM. Deep brain stimulation in 18 patients with severe Gilles de la Tourette syndrome refractory to treatment: The surgery and stimulation. J Neurol Neurosurg Psychiatry 2008; 79: 136-42.

[21] Porta M, Brambilla A, Cavanna AE, Servello D, Sassi M, Rickards H, Robertson MM. Thalamic deep brain stimulation for severe treatment-refractory Tourette Syndrome: Two-year outcome. Neurology 2009; 73: 1375-80.

[22] Servello D, Sassi M, Brambilla A, Defendi S, Porta M. Long-term, post-deep brain stimulation management of a series of 36 patients affected with refractory Gilles de la Tourette syndrome. Neuromodulation 2010; 13: 187-194.

[23] Vernaleken I, Kuhn J, Lenartz D, Raptis M, Huff W, Janouschek H, Neuner I, Schaefer WM, Gründer G, Sturm V. Bithalamical deep brain stimulation in Tourette syndrome is associated with reduction in dopaminergic transmission. Biol Psychiatry 2009; 66: e15-7.

[24] Vernaleken I, Kuhn J, Janouschek H, et al. Acute and chronic effects of bithalamic deep brain stimulation on dopaminergic transmission. NeuroImage 2010; 52(S1): S103.

[25] Houeto JL, Karachi C, Mallet L, Pillon B, Yelnik J, Mesnage V, Welter ML, Navarro S, Pelissolo A, Damier P, Pidoux B, Dormont D, Cornu P, Agid Y. Tourette’s syndrome and deep brain stimulation. J Neurol Neurosurg Psychiatry 2005; 76: 992-5.

[26] Vilela Filho O, Ragazzo PC, Silva DJ, Souza JT, Oliveira PM, Ribeiro TMC. Bilateral Globus Pallidus Externus Deep Brain Stimulation (GPe-DBS) for the Treatment of Tourette Syndrome: An Ongoing Prospective Controlled Study (Abstr.). Stereotact Funct Neurosurg 2007; 85: 42-3.

[27] Dueck A, Wolters A, Wunsch K, Bohne-Suraj S, Mueller JU, Haessler F, Benecke R, Buchmann J. Deep brain stimulation of globus pallidus internus in a 16-year-old boy with severe Tourette syndrome and mental retardation. Neuropediatrics 2009; 40: 239-42.

[28] Foltynie T, Martinez-Torres I, Zrinzo L, Joyce E, Cavanna A, Jahanshahi M, Limousin P, Hariz M. Improvement in vocal and motor tics following DBS of motor GPi for Tourette syndrome, not accompanied by subjective improvement in quality of life – A case report. Mov Dis 2009; 24: S497-8.

[29] Dehning S, Mehrkens JH, Müller N, Bötzel K. Therapy-refractory Tourette syndrome: beneficial outcome with globus pallidus internus deep brain stimulation. Mov Disord 2008; 23: 1300-2.
[30] Shahed J, Poysky J, Kenney C, Simpson R, Jankovic J. GPi deep brain stimulation for Tourette syndrome improves tics and psychiatric co-morbidities. Neurology 2007; 68: 159-60.
[31] Gallagher CL, Garell PC, Montgomery EB. Hemi tics and deep brain stimulation. Neurology 2006; 66: E12.
[32] Diederich NJ, Kalteis K, Stamenkovic M, Pieri V, Alesch F. Efficient internal pallidal stimulation in Gilles de la Tourette syndrome: A case report. Mov Disord 2005; 20: 1496-9.
[33] Van der Linden C, Colle H, Vandewalle V, Alessi G, Rijckaert D, De Waele L. Successful treatment of tics with bilateral internal pallidum (GPi) stimulation in a 27-year-old male patient with Gilles de la Tourette's syndrome. Mov Disord 2002; 17; S341.
[34] Neuner I, Podoll K, Lenartz D, Sturm V, Schneider F. Deep brain stimulation in the nucleus accumbens for intractable Tourette's syndrome: Follow-up of 36 months. Biol Psychiatry 2009; 65: 5-6.
[35] Zabek M, Sobstyl M, Koziara H, Dzierzecki S. Deep brain stimulation of the right nucleus accumbens in a patient with Tourette syndrome. Case report. Neurol Neurochir Pol 2008; 42: 554-9.
[36] Kuhn J, Lenartz D, Mai JK, Huff W, Lee SH, Koulousakis A, Klosterkoetter J, Sturm V. Deep brain stimulation of the nucleus accumbens and the internal capsule in therapeutically refractory Tourette-syndrome. J Neurol 2007; 254: 963-5.
[37] Servello D, Sassi M, Brambilla A, Porta M, Haq I, Foote KD, Okun MS. De novo and rescue DBS leads for refractory Tourette syndrome patients with severe co-morbid OCD: A multiple case report. J Neurol 2009; 256: 1533-9.
[38] Burdick A, Foote KD, Goodman W, Ward HE, Ricciuti N, Murphy T, Haq I, Okun MS. Lack of benefit of accumbens/capsular deep brain stimulation in a patient with both tics and obsessive-compulsive disorder. Neurocase 2010; 22: 1-10.
[39] Martinez- Torres I, Hariz MI, Zrinzo L, Foltynie T, Limousin P. Improvement of tics after subthalamic nucleus deep brain stimulation. Neurology 2009; 72: 1787-9.
[40] Pansaon Piedad JC, Rickards HE, Cavanna AE. What Patients with Gilles de la Tourette Syndrome Should be Treated with Deep Brain Stimulation and What is the Best Target? Neurosurgery 2012.
[41] Groenewegen HJ, van den Heuvel OA, Cath DC, Voorn P, Veltman DJ. Does an imbalance between the dorsal and ventral striatopallidal systems play a role in Tourette's syndrome? A neuronal circuit approach. Brain Dev 2003; 25 Suppl 1: S3-S14.
[42] Ackermans L, Duits A, Temel Y. Long-term outcome of thalamic deep brain stimulation in two patients with Tourette syndrome. J Neurol, Neurosurg, Psychiatry 2010; 81: 1068-7.
[43] Mink JW, Walkup J, Frey KA, Como P, Cath D, DeLong MR, Erenberg G, Juncos J, Leckman JF, Swerdlow N, Visser-Vandewalle V, Vitek JL for the Tourette Syndrome Association, Inc. Recommended Guidelines for Deep Brain Stimulation in Tourette Syndrome. Mov Disord.
[44] Visser-Vandewalle V, Van der Linden Ch, Ackermans L, Temel Y, Tijssen MA, Schruers K, Nederveen P, Kleijers M, Boon P. Deep brain stimulation in Gilles de la Tourette‘s syndrome. Guidelines of the Dutch-Flemish Tourette Surgery Study Group. Neurosurgery 2006; 58: E590.
[45] Müller-Vahl KR, Cath DC, Cavanna AE, Dehning S, Porta M, Robertson MM, Visser-Vandewalle V; ESSTS Guidelines Group. European clinical guidelines for Tourette syndrome and other tic disorders. Part IV: Deep brain stimulation. Eur Child Adolesc Psychiatry 2011; 20: 209-17.
[46] Leckman JF, Riddle MA, Hardin MT, Ort SI, Swartz KL, Stevenson J, Cohen DJ. The Yale Global Tic Severity Scale: Initial testing of a clinician-rated scale of tic severity. J Am Acad Child Adolesc Psychiatry 1989; 28: 566-73.
[47] Ackermans L, Temel Y, Bauer NJC, Visser-Vandewalle V. Vertical gaze palsy after thalamic stimulation for Tourette syndrome: Case report. Neurosurgery 2007; 61: E1100.
[48] LeVasseur AL, Flanagan JR, Riopelle RJ, Munoz DP. Control of volitional and reflexive saccades in Tourette's syndrome. Brain 2001; 124: 2045-58.
[49] Porta M, Sassi M, Ali F, Cavanna AE, Servello D. Neurosurgical treatment for Gilles de la Tourette syndrome: The Italian perspective. J Psychosom Res 2009; 67: 585-90.
